# Germination Ecology and Seed Dispersal of a Critically Endangered Plant: A Case Study of *Pomaderris vacciniifolia* (Round-Leaf Pomaderris)

**DOI:** 10.1371/journal.pone.0161665

**Published:** 2016-08-24

**Authors:** John Patykowski, Matthew Dell, Maria Gibson

**Affiliations:** 1 Centre for Integrative Ecology, School of Life and Environmental Sciences, Deakin University, Geelong, Victoria, Australia; 2 Ecology Australia, Fairfield, Victoria, Australia; Chinese Academy of Forestry, CHINA

## Abstract

Change in ecosystem disturbance regimes from human land-use poses a worldwide problem for management of rare species. Two important types of disturbance influencing the persistence of species in Australian ecosystems are habitat fragmentation and fire. In this study, seed dispersal and the germination ecology of *Pomaderris vacciniifolia*—a critically endangered, rare endemic Australian shrub—were examined to identify likely influences of fire and fragmentation on the decline of populations. The response of seed germination to simulated effects of wildfire and canopy openings was investigated, as was the unaided dispersal capability of seeds from parent plants. A significant increase in germination rate was observed following 100°C heat treatment to seeds, while smoke and light exposure had little influence. Seed imbibition was strongly influenced by heat treatment. The findings indicate a likely positive post-fire germination response, with implications for recruitment success determined by moisture availability following fire. Unaided seed dispersal was limited, which partly explains the apparent decline of populations. Understanding disturbance requirements for threatened species, and subsequent management of landscapes for disturbance, will aid conservation of rare species throughout the world.

## Introduction

Disturbance and habitat fragmentation influences species persistence [1, 2]. Fire acts as a disturbance upon much of the world’s land surface [3] and is a key evolutionary driver selecting for traits which drive the persistence of certain plant taxa [4] and shape plant communities [5, 6]. Fragmentation can result in speciation and endemism through divergent selection and reproductive isolation [7, 8]; however, the persistence of species also can be threatened when critical habitat becomes fragmented [1] or when disturbance regimes to which they are adapted become altered. Rare species, those with limited abundance or restricted distribution [9], are particularly susceptible. For example, the Australian Mallee Emu-wren *Stipiturus mallee* Campbell, a narrow-range endemic, depends on vegetation that has been unburnt for at least 15 years; widespread and over-frequent burning in its habitat has led to the localised extinction of some *S*. *mallee* populations and severe decline in others [10]. Conversely, exclusion of fire in a Finnish boreal forest reduced levels of standing dead wood; reintroducing fire greatly improved saproxylic beetle richness and abundance, particularly for 20 rare species which were found exclusively in recently burnt areas [11]. The decline of many rare plant species from Western Australia, such as Blunt Wattle *Acacia aprica* Maslin & A.R.Chapman, Spiral-fruited Wattle *Acacia cochlocarpa* Meisn. subsp. *cochlocarpa*, and Southern Shy Featherflower *Verticordia fimbrilepis* A.S.George subsp. *fimbrilepis* has been linked to various combinations of reduced fire-frequency, grazing, competition from weeds and land-fragmentation [12, 13]. Although some species in fragmented communities can maintain viable populations through long-distance dispersal between sub-populations and landscapes, this type of dispersal is generally rare in plants [14]. Fragmentation can negatively affect a species’ ability to disperse [15], colonise new areas [16, 17] and cope with climate change through migration [18]. Rare species may have a narrow ecologic amplitude [19] and have few populations to provide insurance against extirpation and stochastic events [20]. Consequently, altered disturbance regimes and habitat fragmentation can lead to the extinction of rare endemic species [21]. Understanding the mechanisms underlying causes of decline in rare species is a critical and fundamental step in effective conservation.

Here we explore the potential influence of changes to disturbance regime and habitat fragmentation on the decline of Round-leaf Pomaderris *Pomaderris vacciniifolia* Reissek (Rhamnaceae), a critically endangered shrub [22] that is endemic to a restricted area of south-eastern Australia [23]. Despite its conservation status, little is known of the ecology of the species and no previous research has been conducted to investigate why it is in decline.

Historical records (pre-2000) indicate that *P*. *vacciniifolia* once was distributed over a much wider range [24, 25]. Landscape changes such as clearing, grazing from native and exotic herbivores, over- and / or under-frequent burning (which also may have depleted the soil seed bank) and competition from weeds all have been implicated as drivers of this decline [25]. The most extensive remaining populations of wild *P*. *vacciniifolia* occur in disjunct and fragmented stands of damp sclerophyll forest in upper catchments of the Yarra River, to the north-east of Melbourne [24]. Most of this area was burnt in fires occurring prior to 1970 [26], which is presumed to have stimulated recruitment in this species [25]. After long inter-fire periods and senescence of mature individuals, pulses of germination were observed for this species in areas that were burnt during fires in 2009, suggesting fire was a main driver of recruitment. However, extant populations occur along roadsides, or where clearing of the overstorey has occurred, thus, access to light also may be an important driver of recruitment.

*Pomaderris vacciniifolia* is a non-serotinous obligate seed regenerator [25] and significant recruitment possibly requires a range of fire related cues to promote germination, as occurs with obligate seed regenerators from other fire prone regions [27]. Seeds which require a specific environmental cue to germinate often have a water impermeable coat as, theoretically, if water enters the coat of a seed requiring specific environmental cues that had not occurred recently, the seed would rot [28]. If seeds do not require specific environmental cues for germination, seed coats are permeable and germination occurs upon watering. Fire can influence seed germination through heating of the seed which can fracture the seed coat or stimulate the embryo to begin development [28]. Smoke (which contains plant growth regulators such as karrikins) also can stimulate the seed embryo to grow [29]. Further, smoke and heat can work together to have synergistic or additive effects on seed germination [30, 31]. Reduced canopy cover from fire or landslip leads to increased solar radiation which also can stimulate seed to germinate [28, 32]. Thus, if the seeds of *P*. *vacciniifolia* are water-impermeable and require specific cues of heat, smoke or light for germination, then changes to the disturbance regime within its natural range may explain why it is rare and in decline.

Obligate seed regenerators rely on the dispersal of seeds to colonise suitable habitat [33], and have evolved a range of traits to accomplish this task. Myrmecochorous dispersal is common for *Pomaderris* species [34] and indeed, *P*. *vacciniifolia* seeds possess elaiosomes [25]. Myrmecochorous dispersal is usually limited to short distances; in the southern hemisphere, mean ant-mediated dispersal distances have been calculated at 1.25 m [35]. As the habitat within the natural range of *P*. *vacciniifolia* is highly fragmented, a second factor potentially contributing to decline in populations may be an inability of seeds to disperse into suitable habitat that is nearby.

This study examined the effect of heat, smoke and light on germination of fresh *P*. *vacciniifolia* seed and determined *in situ* seed dispersal distances. The potential influence of fire regimes and habitat fragmentation on the distribution of *P*. *vacciniifolia* is discussed, along with management recommendations for its long term conservation.

## Methods

*Pomaderris vacciniifolia* is protected by the State Government of Victoria, Australia as a threatened species under the *Flora and Fauna Guarantee Act* 1988. A permit to conduct research on public land and take reproductive material of *P*. *vacciniifolia* under the *National Parks Act* 1975 and the *Flora and Fauna Guarantee Act* 1988 was issued for this study by the Victorian Government Department of Sustainability and Environment (Permit number 10006181). Authorisation to access and conduct research on land managed by Parks Victoria was granted by the relevant Ranger-in-Charge, and permission to access and conduct research on private land was granted by the relevant land-owner. This species became protected as a critically endangered species under the Australian Government *Environment Protection and Biodiversity Conservation Act* 1999 in 2014, after this study was completed.

### Study area

Ten populations of *P*. *vacciniifolia*, each consisting of approximately 15–50 reproductively mature individuals, were located based on historical records [24] and used for sampling in this study. Regional climate data recorded between 1953 and 2006 by the Bureau of Meteorology [36] at the Toolangi (Mt St Leonard) weather station shows mean annual temperature was 15.8°C, with the warmest month being February (23.2°C), and the coolest month July (8.6°C). Rainfall data collected between 1953 and 2011 indicated annual mean monthly rainfall was 113.55 mm, with the driest month being February (76 mm), and the wettest month September (137.9 mm).

### Seed collection and selection

Seeds of *P*. *vacciniifolia* were collected from 60 plants across 10 populations, with six plants randomly selected from each population. Seeds were collected in bags secured over maturing infructescences and left *in situ* until the fruits had matured and released their seeds. Properly developed seed possessed a hard, glossy black seed coat. Underdeveloped and presumably non-viable seeds, which were soft and/or hollow, were discarded. Seeds were stored in dry, dark conditions at room temperature until used in experimentation.

### Seed coat permeability

Water permeability of the seed coat was tested by measuring water uptake over time [28]. Five hundred seeds were randomly selected and, due to their small size, divided into ten replicates of 50 seeds. Five of these replicates were randomly chosen and individually heated at 100°C for five minutes in a fan-forced oven; the other five replicates were left unheated as a control. Each replicate was weighed and then placed between filter paper moistened with room-temperature distilled water. Each replicate of 50 seeds was removed at hourly intervals, blotted dry and re-weighed over a six-hour period, then once daily for 11 days, and once more after 14 days. An increase in weight over this time indicates that water has entered the seed coat and thus the seeds are permeable to water.

### Germination cue tests

Germination cue tests were adapted from the work of Thomas et al. [31]. Seeds were treated with combinations of heat (unheated control, 50°C or 100°C for five minutes), smoke exposure (unsmoked control, 10 mins or 20 mins of aerosol smoke), and light exposure (12,400 lux—moderate sun, 1000 lux—below forest canopy or <0.01 lux—top 10 mm of soil). Light levels were designed to mimic natural conditions of disturbance (no forest canopy), no disturbance (forest canopy intact) and those of buried seed respectively. Each of the 27 treatment combinations was applied to five replicates, each of 50 seeds.

Seeds were heat treated by placing each replicate in an individual, open glass petri dish into a fan-forced oven for five minutes at the required temperatures; each dish of seeds being heated individually [37]. Our aim was to determine whether a germination response to heat occurred, thus, we opted to include a 50°C treatment to reflect sub-soil temperatures of deeply buried seed, and a 100°C treatment to simulate temperatures of seed closer to the surface [38, 39]. Treatments between 50–100°C were not included, as seed from this critically endangered species was limited, and temperatures within this range are known to break seed dormancy and stimulate germination from the soil seed bank [28]. Temperatures beyond 100°C often can lead to seed mortality [40, 41], thus, heat-treatments above 100°C were excluded due to limited seed and a high likelihood it would result in seed mortality.

Smoke treatments (unsmoked control, 10 mins or 20 mins aerosol smoke) were applied to seeds by burning dry, fine fuel litter in a bee smoker. The litter was collected from the vicinity of *P*. *vacciniifolia* populations and was composed predominantly of *Eucalyptus* species leaves and twigs. Smoke from the bee smoker was forced through a condensing tube to cool and dry the smoke, into a chamber containing the seeds, each replicate being smoked individually [37]. For seeds that received both heat shock and smoke treatments, heat shock was applied first.

Treated seeds were transferred to plastic, 9 cm diameter petri dishes, placed on a single layer of Whatman Number 1 filter paper and moistened with distilled water. These were then placed in a growth chamber, where 24 hour lighting was controlled using four GroLux T8 15 Watt fluorescent tubes, and temperatures were kept at a constant 25°C.

Seeds were kept moist and checked weekly for germination. A seed with a 1 mm long radicle was recorded as having germinated and then removed from the sample. Each petri dish was left in the growth chamber for three months, until no further seeds germinated over a two week period.

### Soil seed bank sampling

Samples of top soil were collected from four populations of *P*. *vacciniifolia*. These populations included specimens that were more than five years old and were chosen to ensure the likelihood that each plant had experienced at least one season of flowering, and thus was likely to have contributed seed to the soil seed bank. Samples were taken from eleven points along transect lines extending outward from the base of five plants per population. These were at the immediate base of each plant, at the midpoint between the plant base and the drip line of the foliage, from the drip line of the foliage and then at distances of 0.5, 1, 2, 3, 4, 5, 7 and 10 m from the drip line of the foliage. Approximately 282 cm^-3^ of topsoil was collected per sample by using a metal cylinder, 6 cm in diameter and 10 cm deep as a guide. Transects were positioned to ensure that no other specimen of *P*. *vacciniifolia* was within 20 m of each transect, to avoid the likelihood of seed from more than one individual being included in the samples.

### Seed preparation and germination

Soil samples were sieved through a 2.5 mm mesh to remove larger seeds and debris, and then through a 0.2 mm mesh to remove clay and smaller particles [42]. The remaining concentrated mix of seeds was then air dried overnight and prepared for germination testing the following day. To promote germination, each sample was immersed in freshly boiled water for 5 mins to provide a heat treatment, strained and left to dry. Although germination tests were running concurrently, it was presumed that heat would promote germination as heating had increased seed-coat permeability in a previous test. Each sample was spread in a layer no greater than 5 mm thick over a punnet of sterilised potting mix. The punnets were bottom irrigated until soil was moist and then placed in a growth cabinet. Conditions were controlled to a minimum of 8°C and a maximum of 26°C, with a 16 hour light, 8 hour dark photoperiod [43]. Temperature fluctuations were synchronised to mimic day and night temperature shifts. Samples were checked weekly and any *P*. *vacciniifolia* germinants were recorded and removed from the sample. Seeds of other species germinating from the sample were removed as soon as they were identifiable as non-target species. Irrigation was checked daily to ensure samples did not become dry.

Germination was allowed to continue for two months, until no new germinants were recorded over a one week period. Irrigation was then ceased until samples had dried. Samples were then agitated to disturb the soil, as similar studies [42, 43] showed this promoted germination of seeds that initially failed to germinate. Samples again were irrigated and checked for new germinants for a further month, during which time no new germinants were recorded.

### Analysis

All data analysis was undertaken using R version 3.2.1 [44]. A trend analysis was undertaken to observe change over time in mean seed weight after soaking in water. Germination cue tests were analysed with Generalised Linear Models in the package ‘MuMIn’ [45]. The effect of light on germination was first tested separately as a random factor as it was least expected to influence germination. It was found to have no effect, and was excluded from further analysis to ensure the most parsimonious model was selected. The effects of smoke and heat on germination were then tested as separate, combined, and interacting factors against the null model, and model selection undertaken using Akaike’s information criterion.

Dispersal distances of seed from parent plants (mean germination events) were compared using a Generalised Additive Mixed Model in the package ‘nlme’ [46] using Poisson distribution, and individual shrub as a random factor. Samples collected from the base of parent plants, from the mid-point between the base of the plant and foliage drip-line, and from drip-line of foliage were combined to represent 0 m.

## Results

### Seed coat permeability

**Fig 1:**
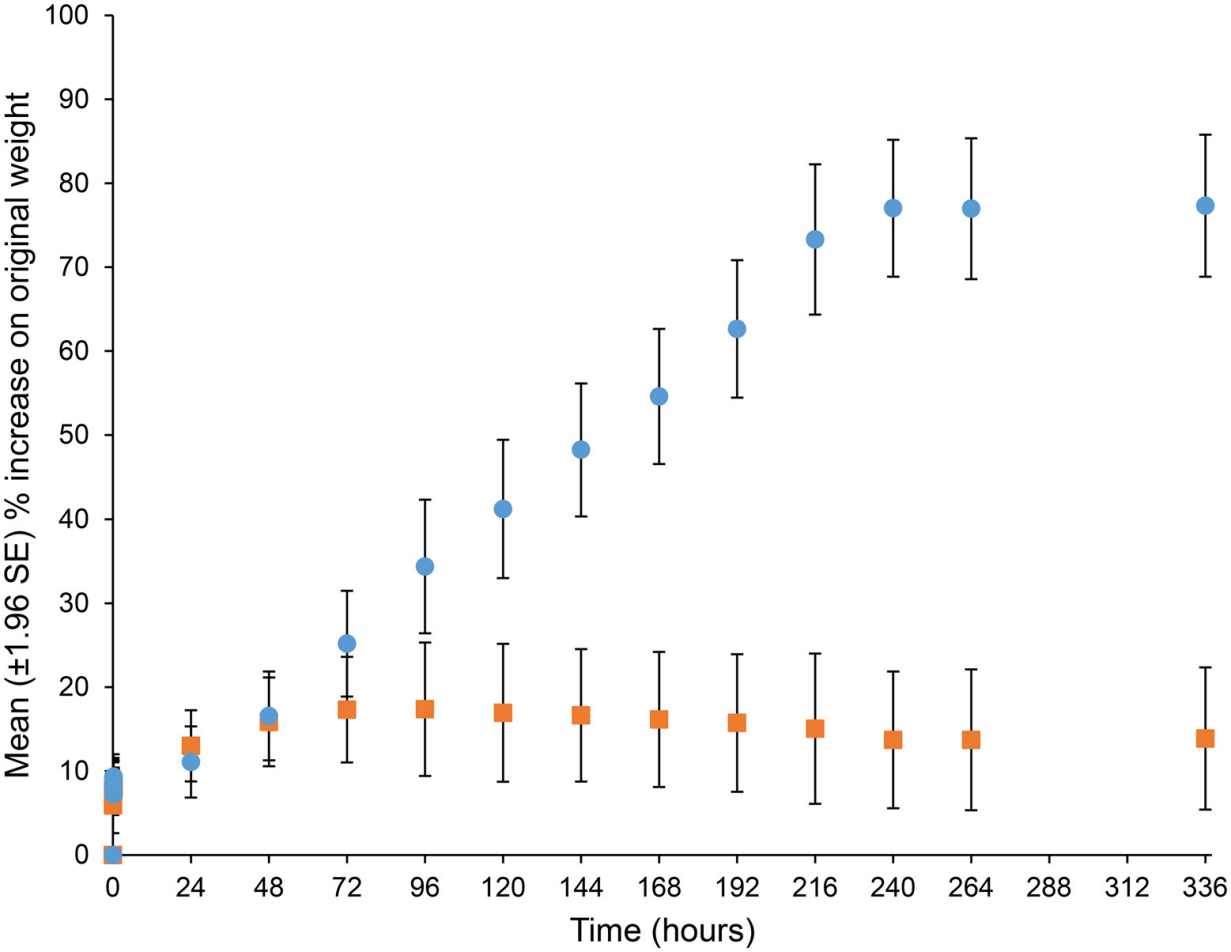
Change in weight of *Pomaderris vacciniifolia* seeds after soaking in water. Circles = heat treated seeds; squares = unheated seeds.

Without a heat treatment simulating fire, seed coats generally remained impermeable to water, i.e. did not imbibe water. Seeds that were heated to 100°C for five minutes exhibited greater imbibition than unheated seeds (Fig 1). Weight increase in heated seeds plateaued after 240 hours of soaking, with a mean increase of 77 ± 1.99% above their original weight. By comparison, unheated seeds exhibited a mean increase of 13.7 ± 8.14% increase.

### Seed germination experiments

**Fig 2:**
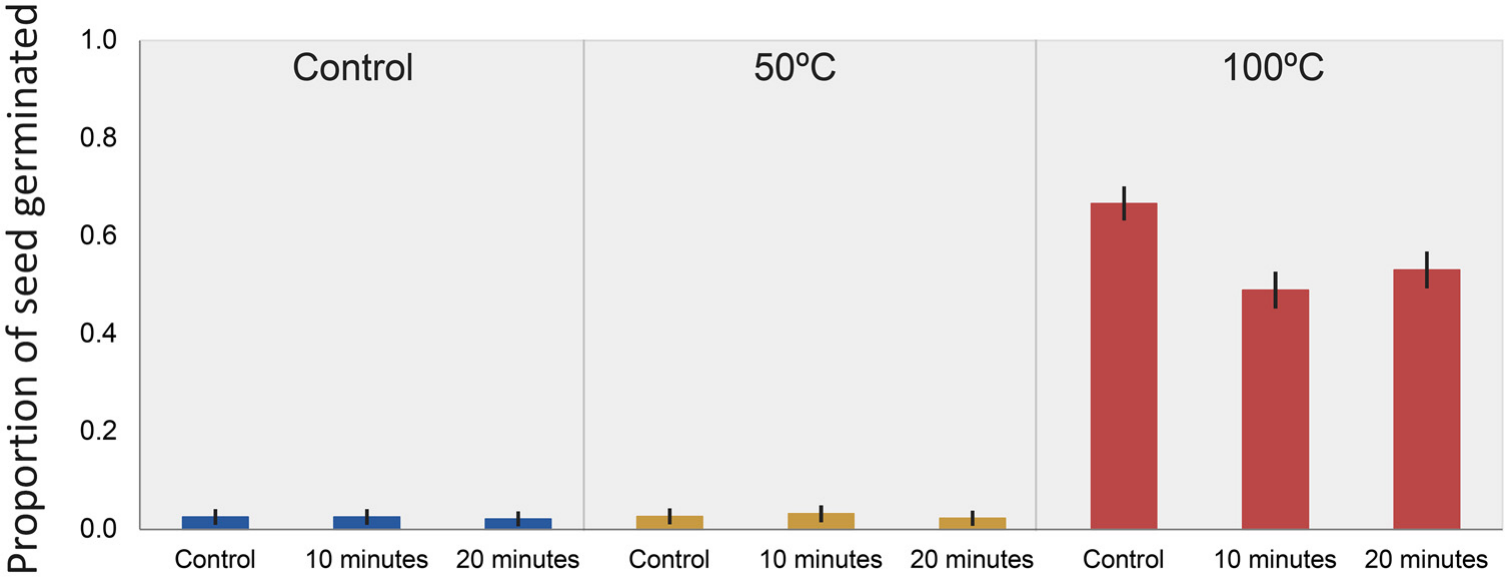
Proportion of *Pomaderris vacciniifolia* seed (± 1.96 SE) germinating after exposure to combinations of heat and smoke. Heat = control, 50°C, 100°C; smoke = control, 10 minutes, 20 minutes.

The model selected as best explaining seed germination included both heat and smoke, with a possible interaction (Table 1). The strength of model fit (*D*^*2*^ = 0.94) explains much of the variance in probability of germination. Parameter estimates (Table 2) indicate that the strongest germination response was observed in seeds that received a heat treatment of 100°C for five minutes (Fig 2), with a less prominent role of smoke. Thus, although smoke is less influential, it does provide some additional information about the probability of germination.

**Table 1:** Models explaining the probability of germination of *Pomaderris vacciniifolia* seeds in relation to combinations of heat and smoke treatments.

Model structure	df	logLik	QAICc	Δ_i_	*w*_i_	*D*^*2*^
**Heat + smoke**	**5**	**-271.05**	**374.60**	**0.00**	**0.67**	**0.93**
**Heat + smoke + (heat:smoke)**	**9**	**-265.29**	**376.00**	**1.43**	**0.33**	**0.94**
Heat	3	-292.68	399.10	24.53	0.00	0.92
Smoke	3	-1621.59	2173.70	1799.10	0.00	0.01
Null model	1	-1634.46	2186.70	1812.08	0.00	0.00

**Table 2:** Parameter estimates from a GLM explaining the relationship between heat and smoke treatments and the germination of *Pomaderris vacciniifolia* seeds.

Variable	Coefficient	SE	z value
Intercept	-3.468813	0.25264	13.633
Heat (50°C)	0.101435	0.29853	0.337
**Heat (100°C)**	**4.108049**	**0.27871**	**14.648**
Smoke (10 minutes)	-0.392628	0.37135	1.053
Smoke (20 minutes)	-0.385704	0.29793	1.285
Heat (50°C): smoke (10 minutes)	0.187816	0.5506	0.338
Heat (100°C): smoke (10 minutes)	-0.73582	0.42277	1.724
Heat (50°C): smoke (20 minutes)	0.009326	0.58618	0.016
Heat (100°C): smoke(20 minutes)	-0.394381	0.43992	0.888

### Seed dispersal

**Fig 3:**
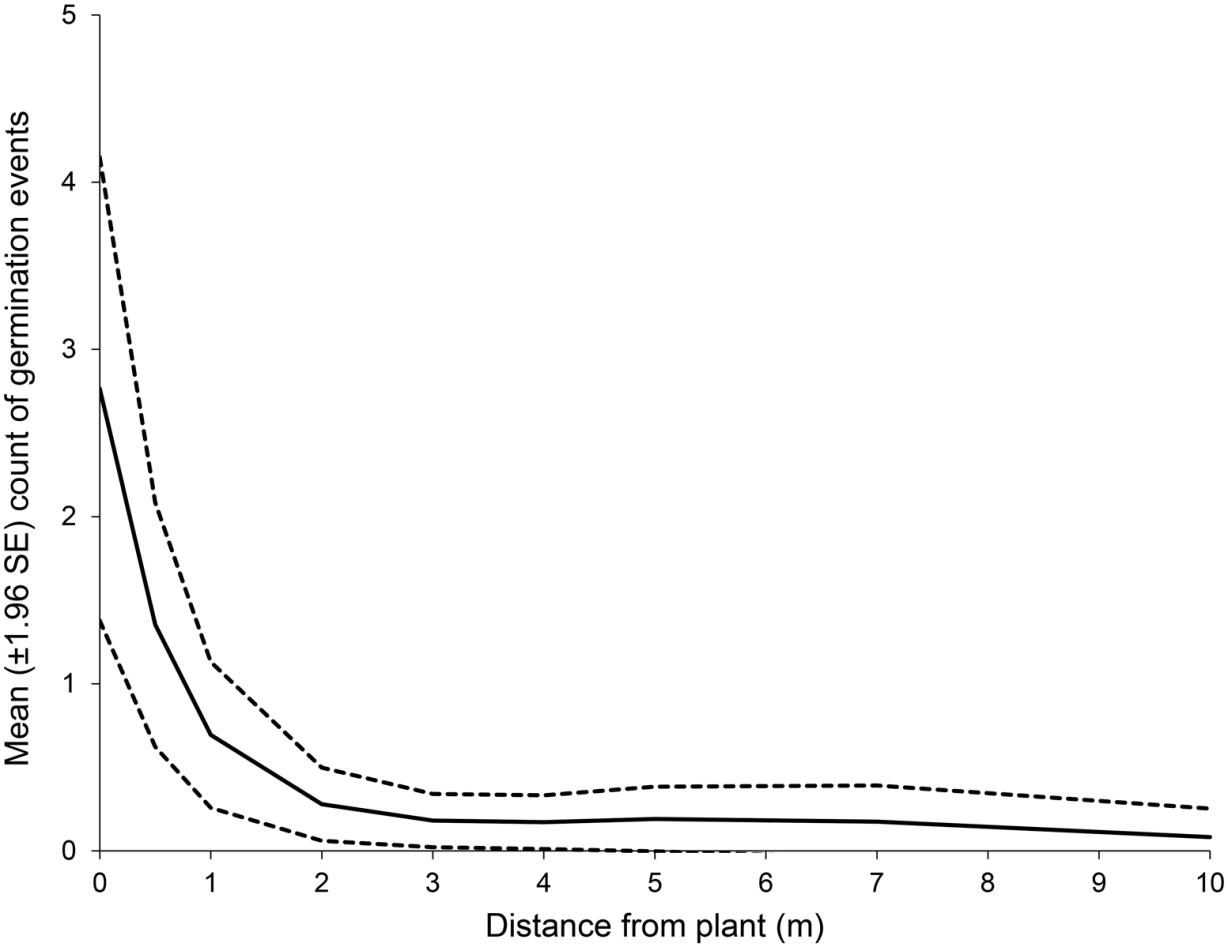
Relationship between mean count of germinated *Pomaderris vacciniifolia* seeds and the distance of the sample from the drip-line of the parent plant’s foliage. Solid line = mean; dashed line = 95% confidence intervals of standard error of mean.

The density of *P*. *vacciniifolia* seed in the soil seed bank was greatest between the base of the parent plant and 0.5 m from the drip-line of its foliage, and densities beyond this distance were negligible (F = 24.3, p<0.001) (Fig 3).

## Discussion

The germination responses of *Pomaderris vacciniifolia* to heat, light and smoke treatment have been determined for the first time. The results provide high quality data for strategic management of threatened species, including documenting traits for ecological burning.

The observed increases in weight of *P*. *vacciniifolia* seed heated to 100°C showed that heating allowed water imbibition through an otherwise water-impervious seed coat. This indicates that an environmental cue is required for germination [28, 47, 48] and that wetting of seed following heat treatement is important for recruitment success. The same observation has been seen in several other Rhamnaceae species [49] suggesting an environmental cue may be required for significant germination events in other species within this family.

Mass germination occurred after a heat treatment of 100°C, suggesting that temperatures likely to be experienced during fire act as an environmental cue for a pulse of seed germination [28]. The 50°C heat treatment reflected soil temperatures of a low intensity fire, and did not result in germination that differed from the control. Temperatures above 50°C are not typically reached in soil below 4 cm during low-intensity fuel reduction burns [39]. Thus, fire intensity and seed burial depth are important factors influencing germination. Low-intensity ecological or fuel reduction burns may fail to stimulate germination if seed is buried too deeply in the soil; high-intensity burns may lead to seed mortality if seed is located too close to the surface. The interaction between typical seed burial depth, fire intensity, soil heating and germination in this species thus requires further attention.

Smoke had a slight negative influence on germination, which is not uncommon [50], although many Australian species germinate with a strong response to smoke or an interaction between smoke and heat [30, 31]. Duration or concentration of smoke exposure previously has been identified as a factor that can inhibit germination [29, 51]. Some species exhibit reduced germination as smoke exposure increases, or an increase in germination as smoke exposure increases but an overall reduction in germination compared to unexposed seeds [30, 31]; this latter pattern is consistent with this study.

These results can, in part, explain why *P*. *vacciniifolia* is threatened with extinction. If fire intervals are greater than ≈ 40 years, the estimated life span of *P*. *vacciniifolia* [25], a contraction in its distribution would occur as recruitment would be minimal; although a soil seed bank may persist. Over-frequent burning (fire intervals < 2 years) would likely deplete the soil seed bank, as this is the minimum time observed for a *P*. *vacciniifolia* germinant to reach maturity and set seed [25]. The influence of heat on seed germination supports the use of ecological burning to increase population sizes and area of occupancy, but an appropriate fire regime must be considered including season and rainfall considerations.

Of seeds exposed to the 50°C and to the control heat treatments, up to 10% (mean 2.65% ± 2.40%) germinated under the growing conditions, including the unheated control, suggesting some recruitment can occur without environmental cues. Water imbibition would be expected in these seeds (Baskin and Baskin 1998), which may explain the small increase in weight of unheated seed replicates. Thus, there is evidence that a small proportion of seeds germinate in the absence of fire. All limiting factors aside, this would ensure that there is the potential for recruitment during inter-fire periods. Senescent plants are replaced with recruits that can provide diaspores to the soil seed bank, ensuring that populations could persist, albeit in small numbers, in the long term absence of fire. Mass germination follows heat shock, which seeds would experience during a fire, leading to greater population sizes, replenishment of the soil seed bank and greater resilience to impacts such as grazing. This recruitment strategy has been recognised for other post-fire recruiters [52, 53]. Maturation of recruits germinating in the absence of fire may be affected by native and exotic herbivores, or competition from native and exotic plants; these factors may threaten populations persisting where fire has been absent, although this needs further attention.

Seed density observations show that seed dispersal distance is limited in *P*. *vacciniifolia*. Although dispersal distances of 0.5 m have been observed as the result of myrmecochory [35], it is more likely that dispersal of *P*. *vacciniifolia* seed is largely unaided, and any passive dispersal is insignificant. We suggest that habitat fragmentation from land clearing, residential development and roads isolate populations of *P*. *vacciniifolia* within its current range, and act as barriers for migration as dispersal distances are limited. Where local extirpation occurs, surrounding populations are highly unlikely to disperse seeds the distances necessary to reach areas of suitable habitat amongst fragmented landscapes, leading to an absence of *P*. *vacciniifolia* from parts of its original range. Mass recruitment in previously colonised areas may occur after fire if there is a viable soil seed bank but currently the longevity of seed is unknown. Reintroducing this species into suitable habitat may be a viable option for replacing populations where ecological burning is inappropriate due to risk to human or ecological assets, or where the soil seedbank has been depleted.

## Conclusion

Reduced fire-frequency and increased habitat fragmentation are likely counterparts in the decline of the nationally threatened *P*. *vacciniifolia*. Seeds require a heat treatment consistent with soil temperatures experienced during bushfire for mass germination. *Pomaderris vacciniifolia* has a limited seed dispersal capability and thus is unlikely to disperse unaided through fragmented landscapes.

Patterns of species decline from changing disturbance regimes are evident for flora and fauna in a range of global contexts [10–13], where multiple disturbance factors may threaten the persistence of rare endemic species. As rare species are more susceptible to extinction than common species [54], it is important that we fully appreciate underlying mechanisms of persistence. Investment in managing ecosystems for disturbance will help maintain biodiversity values in ecological systems and aid the conservation of rare species.
